# Selective Adsorption of Ionic Species Using Macroporous Monodispersed Polyethylene Glycol Diacrylate/Acrylic Acid Microgels with Tunable Negative Charge

**DOI:** 10.3390/gels9110849

**Published:** 2023-10-26

**Authors:** Minjun Chen, Ksenija R. Kumrić, Conner Thacker, Radivoje Prodanović, Guido Bolognesi, Goran T. Vladisavljević

**Affiliations:** 1 Department of Chemical Engineering, Loughborough University, Loughborough LE11 3TU, UK; m.chen2@lboro.ac.uk (M.C.); g.bolognesi@ucl.ac.uk (G.B.); 2 Laboratory of Physics, Vinča Institute of Nuclear Sciences—National Institute of the Republic of Serbia, University of Belgrade, 11001 Belgrade, Serbia; 3 Department of Materials, Loughborough University, Loughborough LE11 3TU, UK; c.j.thacker-20@student.ac.uk; 4 Faculty of Chemistry, University of Belgrade, Studentski Trg 12-16, 11000 Belgrade, Serbia; rprodano@chem.bg.ac.rs; 5 Department of Chemistry, University College London, London WC1H 0AJ, UK

**Keywords:** hydrogels, porous gels, microfluidics, anionic microgels, biocompatible polymers, surface charge, tissue engineering, copolymer hydrogels, PEGDA, PEGDA–AA hydrogels

## Abstract

Monodispersed polyethylene glycol diacrylate (PEGDA)/acrylic acid (AA) microgels with a tuneable negative charge and macroporous internal structure have been produced using a Lego-inspired droplet microfluidic device. The surface charge of microgels was controlled by changing the content of AA in the monomer mixture from zero (for noncharged PEGDA beads) to 4 wt%. The macroporosity of the polymer matrix was introduced by adding 20 wt% of 600-MW polyethylene glycol (PEG) as a porogen material into the monomer mixture. The porogen was successfully leached out with acetone after UV-crosslinking, which resulted in micron-sized cylindrical pores with crater-like morphology, uniformly arranged on the microgel surface. Negatively charged PEGDA/AA beads showed improved adsorption capacity towards positively charged organic dyes (methylene blue and rhodamine B) compared to neutral PEGDA beads and high repulsion of negatively charged dye molecules (methyl orange and congo red). Macroporous microgels showed better adsorption properties than nonporous beads, with a maximum adsorption capacity towards methylene blue of 45 mg/g for macroporous PEGDA/AA microgels at pH 8.6, as compared to 23 mg/g for nonporous PEGDA/AA microgels at the same pH. More than 98% of Cu(II) ions were removed from 50 ppm solution at pH 6.7 using 2.7 mg/mL of macroporous PEGDA/AA microgel. The adsorption of cationic species was significantly improved when pH was increased from 3 to 9 due to a higher degree of ionization of AA monomeric units in the polymer network. The synthesized copolymer beads can be used in drug delivery to achieve improved loading capacity of positively charged therapeutic agents and in tissue engineering, where a negative charge of scaffolds coupled with porous structure can help to achieve improved permeability of high-molecular-weight metabolites and nutrients, and anti-fouling activity against negatively charged species.

## 1. Introduction

Hydrogels are increasingly used as excipients for the controlled release of therapeutics [1], scaffolds for repairing and regenerating human tissues and organs [2], and replacements for tissues and organs [3]. Surface charge is one of the key factors of hydrogel scaffolds in tissue engineering and regenerative medicine applications, since the charge strongly affects the adhesion, spreading, and proliferation of tissue cells [3], differentiation of stem cells [4], and antifouling properties of scaffolds [5]. Ionic groups present in the polymer network of hydrogels can undergo electrostatic interactions with other charged static surfaces and mobile charges in the solution, which can have a profound effect on the swelling behavior of hydrogels, their mechanical properties, and the loading capacity and release kinetics of encapsulated charged drugs [6]. Furthermore, the ionic charge of hydrogels plays a crucial role in mimicking the functional properties of natural tissues and exhibiting strain-stiffening effect [7] and volume deformations in response to electric stimulation mimicking the behavior of skeletal muscle tissues [8].

In addition to surface charge, morphological properties of hydrogels such as size, shape, and porosity are also important for biomedical applications. In this respect, hydrogel microspheres, called microgels, are particularly useful since they can serve as modular constructs in tissue engineering that can be crosslinked to one another to form 3D scaffolds of tunable architecture [9]. Compared to bulk hydrogels, microgels have several advantages, including fast response to external stimuli, high permeability to dissolved oxygen and low-molecular-weight nutrients and metabolic wastes, and injectability, which allows the administration of microgels to patients using minimally invasive procedures [10]. 

Microgels fabricated by droplet microfluidics are gaining in popularity due to the tunable size, shape, and morphology of particles that can be obtained [11] and the ability to encapsulate single cells [12] and achieve a controlled spatial distribution of cells within the particles [13]. Ideally, cell-laden microgels should also be permeable to high-molecular-weight biological molecules to allow unrestricted growth of cells within the polymer network. However, the mesh size of conventional polymer networks is in the nanometer range, typically from a few nm to 100 nm. To improve the permeability of microgels to high-molecular-weight species, macro-pores with a size greater than 1 μm can be formed in the polymer matrix, which also leads to improved water uptake and adsorption capacity. A facile method of introducing macropores into microgels is porogen templating, which provides superior tunability of the pore morphology [14,15]. Porogen is a sacrificial material that occupies the place of a pore while the polymer is synthesized and is leached out after crosslinking to leave the pores behind [15]. Several porogen materials have been tested so far, including organic liquid droplets, ice particles, air bubbles, and salt crystals [16]. 

In this work, we used poly (ethylene glycol) (PEG) as a biodegradable, eco-friendly porogen to introduce macropores in anionic poly (ethylene glycol) diacrylate (PEGDA)/acrylic acid (AA) microgels. For the first time, monodispersed PEGDA/AA microgels with tunable charge and porosity were manufactured using a microfluidic device composed of coaxial glass capillaries and CNC-machined Lego-inspired plastic blocks [17]. Microfluidic devices are usually fabricated by soft lithography [18], etching [19], and 3D printing [20]. However, these devices are usually permanently bonded or even monolithic and difficult to clean and regenerate after use. On the other hand, Lego-inspired glass capillary devices can be taken apart and reassembled like Lego bricks, which means that they can be dismantled after use and each part can be cleaned separately. Also, glass has excellent optical transparency and chemical stability. Recently, Lego glass capillary devices were used for the production of nonporous PEGDA microgels [21] and encapsulation of phase change materials [22]. The production of macroporous PEGDA/AA microgels is a novel application of droplet microfluidic devices. Successful copolymerization between PEGDA and AA was confirmed through adsorption experiments with a series of positively and negatively charged organic dyes and Cu(II) ions. 

## 2. Results and Discussion

### 2.1. Optimisation of Fluid Flow Rates and UV Exposure Time

The continuous phase (CP) contained 3.5 wt% XIAMETER^®^ PMX-200 surfactant to prevent droplet coalescence [21]. When the CP flow rate was too high, jetting of the dispersed phase (DP) occurred due to the high shear rate at the liquid/liquid interface, pulling the DP into a long jet, and droplet production was not possible. In contrast, when the CP flow rate was too low, large globular droplets of varying diameter were produced. The flow rates indicated in Table 1 allowed for stable generation of uniform droplets over at least 180 min. The optimum flow rate ratio Q_c_/Q_d_ was between 2.0 and 3.5 and 6−10 h was needed to produce 1000 mg of microgel particles. Interestingly, the presence of acrylic acid and PEG in the DP allowed for higher droplet throughputs. 

Monomer mixture droplets were cured by off-chip UV polymerization. Figure S1 shows the morphology of PEGDA–PEG–AA(2%) beads after exposing the droplets to UV light (75 mW/cm^2^) for 120, 240, 480, and 600 s. A core-shell morphology was observed for particles exposed to UV radiation for less than 120 s, which indicated that crosslinking was not complete [21]. In fact, oxygen dissolved in silicon oil diffused to the particle surfaces and inhibited polymerization in the surface region [21]. For 240 s and 480 s exposure times, particles had wrinkly, non-spherical morphologies, Figure S1b,c. Often, when a photo-curable monomer is exposed to UV light, wrinkles arise on the surface before the monomer is fully polymerized [23]. Wrinkled surfaces are formed by various mechanisms including temperature gradient, mechanical gradient, swelling [24], and chemical gradient [25]. In this case, wrinkling occurred due to the gradient in crosslinking density within the polymer during curing. Oxygen dissolved in silicon oil diffuses into droplets and inhibits the polymerization, creating a liquid layer of uncured PEGDA on the surface of partially cured droplets. After that, uncured PEGDA molecules in the top layer spontaneously diffuse into and swell the underlying crosslinked PEGDA network, generating the compressive stress that leads to surface buckling. As the degree of crosslinking increased, fewer monomer molecules were left on the surface, which increased the rigidity of the surface and resulted in particles with a smoother surface (Figure S1d). Formulations that contained higher water content required longer polymerization time as the distance between monomer molecules within the droplets was higher, Table 1.

### 2.2. Droplet and Particle Polydispersity

**Figure 1:**
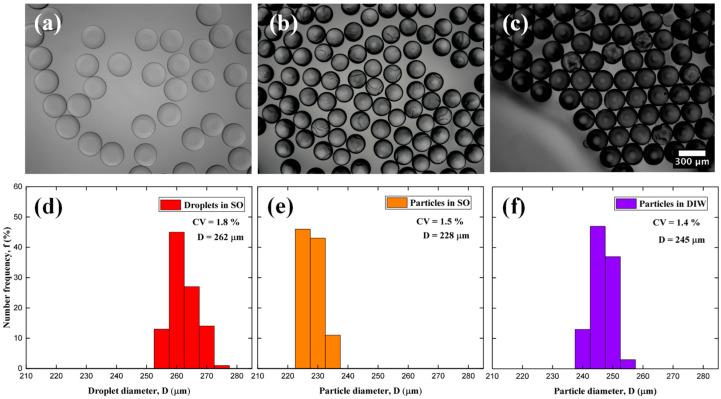
Optical microscopy images and particle size distribution of PEGDA–PEG–AA (2%) sample: (**a**,**d**) Droplets in silicone oil; (**b**,**e**) Microgels in silicone oil; (**c**,**f**) Microgels transferred to DIW.

Many properties of microgels are size-dependent, such as syringeability, filterability, optical properties, agglomeration tendency, rheological properties in suspension, adsorption capacity, and adhesion properties. Control over the particle size allows the behavior of the synthesized beads to be predictable. The particle size distributions of PEGDA–PEG–AA(2%) microgels in different production stages are shown in Figure 1. The droplets were dripped onto a glass slide in a large drop of silicone oil and exposed to UV light at 75 mW/cm^2^ for times shown in Table 1. Using the ‘Oval” tool in ImageJ, the mean droplet and particle diameter could be measured. To establish the polydispersity of particle sizes, the coefficient of variation was used:

(1) $$CV=\frac{\sigma }{D}$$

where σ is the standard deviation and D is the average diameter. For each stage of particle synthesis, CV values of 1–2% were obtained using Equation (1). According to the standards of the National Institute of Standards and Technology, particles are classified as monodisperse as CV < 3% [26]. The average droplet diameter decreased by 13% during polymerization, Figure 1b, and the droplet volume was reduced by 34% compared to the initial droplet volume, which is close to the percentage of DIW in the DP. Therefore, during polymerization, DIW was mainly removed and PEG remained in the particles and contributed to their macroporous structure; then, DIW and PEG were washed away with acetone. When the particles were transferred from silicone oil to DIW, their average diameter and volume increased by 7% and 24%, respectively, due to swelling. Also, the particles become darker after washing since the light from the microscope was scattered by the newly formed pores. 

### 2.3. ATR-FTIR

**Figure 2:**
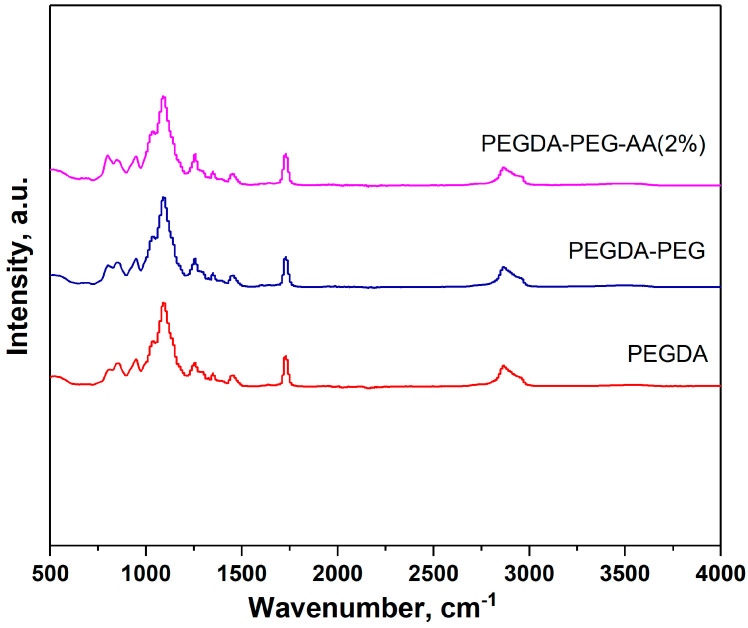
ATR-FTIR spectra of pure PEGDA, PEGDA–AA(2%), and PEGDA–PEG–AA(2%) microgels in the range of 500–4000 cm^−1^.

PEGDA, PEGDA–AA(2%), and PEGDA–PEG–AA(2%) samples were analyzed using ATR-FTIR, Figure 2. However, we could not distinguish between pure PEGDA and PEGDA-AA samples due to the overlap between the characteristic bands in both samples. For example, the C–O stretch appears at 1320–1210 cm^−1,^ and the C–O bond is present in both monomeric units (within ether groups of PEGDA and carboxylic groups of AA). The carbonyl stretch C=O of a carboxylic acid in AA residues appears from 1760 to 1690 cm^−1^ but overlaps with the carbonyl stretch of ester groups in PEGDA residues. In addition, the carboxylic acid O–H stretch appears in the region 3000–2500 cm^−1^, which overlaps with the C–H stretching band. No absorption peak was found above 3000 cm^−1^. Compounds that do not have a C=C bond show C–H stretches only below 3000 cm^−1^, which means that non-polymerized PEGDA molecules were fully removed from microgel particles during washing and C=C double bonds from terminal acrylate groups of uncured PEGDA could not be detected. 

### 2.4. SEM Characterisation

**Figure 3:**
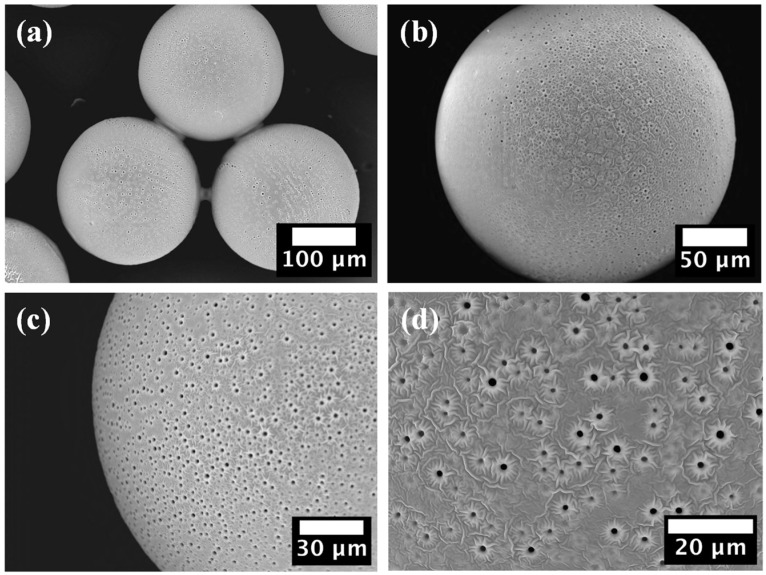
SEM images of PEGDA–PEG–AA(2%) microgel, confirming its macroporous structure: (**a**) Three particles; (**b**) Single particle; (**c**) Close-up on particle edge; (**d**) Close-up of the particle surface.

SEM images of the PEGDA–PEG–AA(2%) microgel sample are shown in Figure 3. 

No cracks or dents were observed on the particle surface that could affect their mechanical stability and physical properties. The presence of cylindrical macro-pores with a diameter of ~1.5 μm is visible at higher magnifications (Figure 3c,d). Closer inspection of the surface revealed a crater-like pore morphology and a floral topography surrounding the pores, Figure 3d. Water and acetone penetrate inside the crosslinked network during washing due to the high affinity of hydrophilic PEGDA chains to water and polar solvents. Crosslinked PEGDA chains are crosslinked and insoluble, while PEG molecules do not have crosslinkable groups but only non-reactive ether bonds. Therefore, PEG molecules were not covalently incorporated in the polymer network and tended to diffuse out of the crosslinked network to establish an osmotic equilibrium with the washing liquid. The osmotic pressure inside the beads can be high due to the high concentration and relatively low molecular weight of PEG. Since reactive monomers formed a dense polymer skin on the surface, there was not enough space between the polymer chains for PEG to escape only by molecular diffusion through the polymer network. As a result, PEG was forcibly ejected through the skin during washing, resulting in tiny craters on the surface. These micron-sized pores can significantly improve the permeability and loading capacity of the microgel since they are three orders of magnitude larger than the mesh size of crosslinked PEGDA (0.1–10 nm) [14]. Also, these pores increase the total surface area of the particles and improve their water absorption ability and adsorption capacity. 

### 2.5. Adsorption of Organic Dyes onto PEGDA and PEGDA–AA Microgels

#### 2.5.1. Adsorption of Cationic Dye (MB) onto Nonporous and Macroporous PEGDA and PEGDA/AA Microgels

Methylene blue (MB) was used as a model cationic compound in all experiments, while other dyes (RhB, CR, MO) and Cu(II) ions were used to provide additional evidence that PEGDA–AA microgels were negatively charged. In these experiments, ~15.5 mg of dry nonporous beads or ~12.5 mg of dry macroporous beads were placed in a series of beakers, each containing 6 mL of MB solution of different concentrations (1–100 ppm) and left for 24 h to allow the system to reach its adsorption equilibrium. A gentle stirring at 150 rpm was applied to prevent microgel settling and accelerate the diffusion of dye molecules to the microgel surface. The equilibrium MB concentration in the solution ($C_{e}$) was measured by spectrophotometric analysis, while the weight of MB adsorbed onto the beads per unit mass of microgel, $q_{e}$ (mg/g) was calculated using Equation (3). 

**Figure 4:**
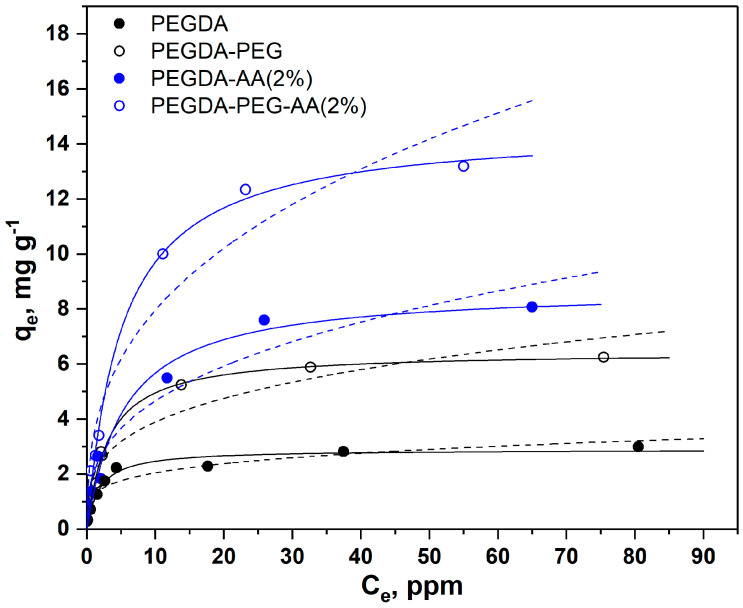
Adsorption isotherms of MB from the aqueous phase (6 mL, pH = 6.3) onto nonporous and macroporous PEGDA and PEGDA/AA microgels (12.5 mg) (solid lines = Langmuir isotherms, and dashed lines = Freundlich isotherms). PEGDA/AA microgels were prepared by adding 2 wt% AA to the dispersed phase.

As shown in Figure 4, the adsorption equilibrium follows the Langmuir model represented by solid lines, while the Freundlich model was not accurate. The Freundlich model provides a good fit to the experimental data if the solid surface is heterogeneous, which causes variations in the heat of adsorption along the surface. However, the dye adsorption process follows the Langmuir model with monolayer adsorption and uniform distribution of active sites at the surface. Both PEGDA/AA microgel samples exhibited higher adsorption capacity towards MB than PEGDA microgels; this is because MB molecules are positively charged at pH 6.3 and attracted by negatively charged carboxylic groups present on AA residues. Depending on pH, MB molecules can be present in the aqueous phase as cations (MB^+^) and undissociated molecules (MB°) [27]. At pH = 2, less than 2% of MB^+^ is present and the adsorption was due to hydrophobic effects. At pH = 3, 14% of MB^+^ is present, while both molecular forms exist in equal quantities at pH = pKa = 3.8. The adsorption experiments were carried out at pH = 6.3, where MB^+^ is virtually the only molecular form of MB present in the aqueous phase. 

The results in Figure 4 also confirm that macroporous microgel particles are more efficient adsorbents than nonporous beads due to the additional surface area of macropores. SEM images in Figure 3 clearly show that micron-sized pores remain in the polymer matrix of macroporous beads after the removal of non-crosslinkable PEG, while non-porous beads have a smooth surface without any macropores. Adsorption is a surface-based process and the adsorption capacity depends on the total surface area available rather than on the total mass. Based on $q_{m}$ values shown in Table 2, macroporous microgels have 1.7–2.2 times higher surface area per unit volume than nonporous microgels. 

The maximum equilibrium MB concentration in the aqueous phase ($C_{e}$) on each curve in Figure 4 is in good correlation with the previous observations. For nonporous PEGDA-AA(2%) beads, the maximum $C_{e}$ value was 65 mg/g, which means that these beads were able to reduce the MB concentration in the liquid phase from 100 ppm to 65 ppm under the applied experimental conditions. Nonporous PEGDA beads were able to reduce the MB concentration in the aqueous phase from 100 ppm to 80 ppm. Macroporous PEGDA-PEG–AA(2%) beads were the most efficient, as shown in Figure 4, and reduced the MB concentration to 55 ppm. The Langmuir and Freundlich parameters calculated from the adsorption data shown in Figure 4 are listed in Table 2. 

As can be seen from Table 2, the maximum adsorption capacity ($q_{m}$) of nonporous PEGDA–AA(2%) beads towards MB was 8.8 mg/g, while the maximum capacity of nonporous PEGDA beads was only 2.9 mg/g, which is three times smaller adsorption capacity. The same trend was observed for macroporous beads, since $q_{m}$ was 15 mg/g for PEGDA–PEG–AA(2%) microgel and only 6.4 mg/g for PEGDA beads. In most cases, the correlation coefficients were higher for the Langmuir isotherm model, indicating that this model more accurately describes the adsorption of MB onto PEGDA-based microgels. 

**Figure 5:**
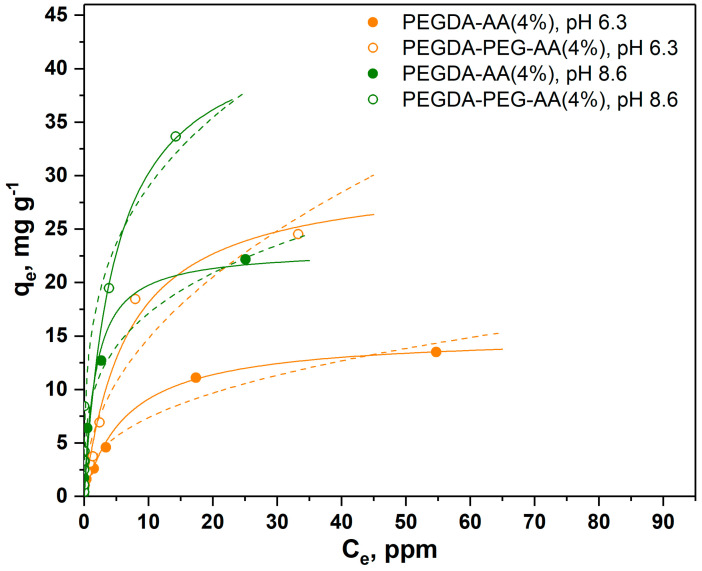
Adsorption isotherms of MB onto nonporous (closed circles) and macroporous (open circles) PEGDA/AA microgels prepared by adding 4 wt% AA to the dispersed phase. The experiments were performed at pH = 6.3 (orange circles) and pH = 8.6 (green circles) (solid lines = Langmuir isotherms, dashed lines = Freundlich isotherms).

In the results shown in Figure 5, a higher amount of AA was added to the dispersed phase (4 wt%) to check if the adsorption equilibrium can be affected by increasing the AA:PEGDA ratio in the dispersed phase. In addition, the experiments were performed at two different pH values. 

As can be seen in Figure 5, the higher amount of AA in the dispersed phase led to higher adsorption capacity of microgels, probably due to the higher amount of carboxylic groups in the polymer network. For example, $q_{m}$ for macroporous microgels increased from 15 mg/g to 30 mg/g when the content of AA in the dispersed phase increased from 2 wt% to 4 wt%. The $q_{m}$ value for nonporous microgels followed the same trend and increased from 8.8 mg/g to 15 mg/g when the content of AA in the dispersed phase increased from 2 wt% to 4 wt%. Also, at the same AA content in the dispersed phase (4 wt%), $q_{m}$ for macroporous microgels was higher at pH = 8.6 (45 mg/g) than at pH = 6.3 (30 mg/g). The trend was the same for nonporous PEGDA/AA microgels, although the adsorption capacity at both pH values was lower (23 and 15 mg/g at pH = 8.6 and pH = 6.3, respectively) than for macroporous beads. The higher adsorption capacity of PEGDA/AA microgels at higher pH values can be explained by the higher percentage of deprotonated carboxylic groups at higher pH, leading to more negative charges on the microgel surface and higher electrostatic attraction of positively charged MB molecules. 

**Figure 6:**
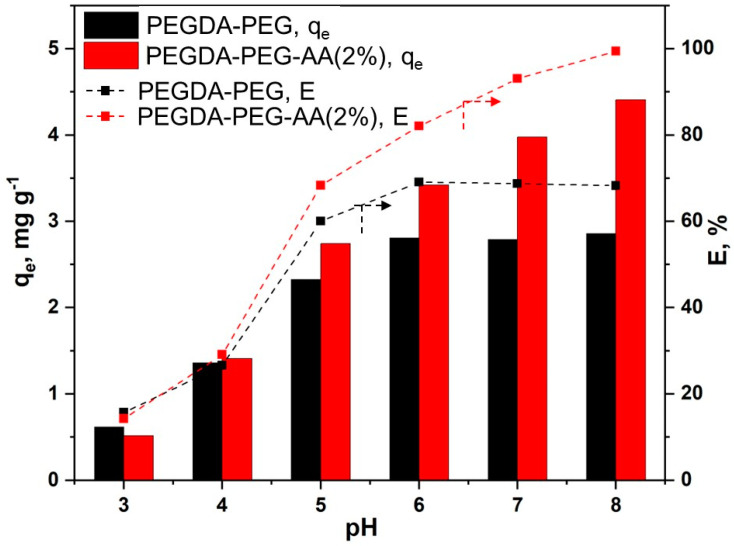
The effects of the pH of MB solution (6 mL) on the equilibrium adsorption capacity and removal efficiency of MB using macroporous PEGDA and PEGDA/AA microgels (12.5 mg). The initial MB concentration in the liquid phase was 10 ppm and the adsorption time was 24 h.

To systematically investigate the influence of pH on the adsorption capacity of macroporous PEGDA and PEGDA/AA microgels, about 12.5 mg of dry microgel particles were placed in 6 mL of MB solution (10 ppm) and the pH was adjusted to 3, 4, 5, 6, 7, and 8. The dispersion was kept under agitation for 24 h to establish an adsorption equilibrium. In each case, the equilibrium MB concentration in the liquid phase was measured and used to calculate the equilibrium removal efficiency (E) and adsorption capacity ($q_{e}$) using Equations (2) and (3), respectively. The results are summarised in Figure 6. 

The solution pH has a twofold effect on the adsorption of MB by PEGDA/AA microgels. Firstly, at pH < 6, MB is not fully charged, and the lower the pH, the lower the percentage of charged MB molecules. Secondly, the pH has a high impact on the charge of polyacrylic acid (PAA) in the pH range of 3−11. PAA becomes increasingly more charged if the pH is increased from 3 to 11 due to the dissociation of carboxylic groups [28]. At pH = 3, the adsorption capacity of both microgels is at minimum (Figure 6), since PEGDA/AA beads are noncharged, there is no electrostatic effect and the dye is adsorbed due to hydrophobic effects. In addition, at pH 3, a great majority of MB molecules are present in neutral form, and this form is generally more poorly adsorbed onto different adsorbents than MB^+^ form [29]. At pH = 4, only 2% of carboxylic groups were charged [28]; thus, there was hardly any charge on PEGDA/AA microgels. As a result, no difference in the adsorption capacity of PEGDA and PEGDA/AA microgels was observed. However, the amount of MB adsorbed onto both microgels more than doubled when the pH rose from 3 to 4. It can be explained by the higher percentage of MB^+^ species in the solution at pH 4 (62%) than at pH 3 (14%). Starting from pH = 5 upwards, the difference in adsorption capacity between PEGDA and PEGDA/AA beads is increasingly significant, due to the exponential increase of the charge of PEGDA/AA microgels. For instance, at pH = 5, the fraction of charged carboxylic groups is 6% but increases to 15% at pH = 6 [27]. At pH = 8, more than 50% of carboxylic groups is charged [27]. For pure PEGDA beads, the adsorption capacity increased up to pH 6 and then remained constant with a further increase in pH, since the fraction of MB^+^ in the solution reached 100% at about pH = 6. The adsorption capacity of PEGDA/AA beads increased up to pH = 8 since the surface of PAA is fully charged only at pH 11. It is important to note that at pH > 8, weak hydrolysis of ester bonds in PEGDA molecules occurs [30] and therefore, the maximum pH applied in this study was 8. Under the investigated conditions, the MB removal efficiency of porous PEGDA–AA microgel reached 100% at pH = 8, while the maximum MB removal efficiency of porous PEGDA microgel was less than 70% and could not be improved at pH > 6.

**Figure 7:**
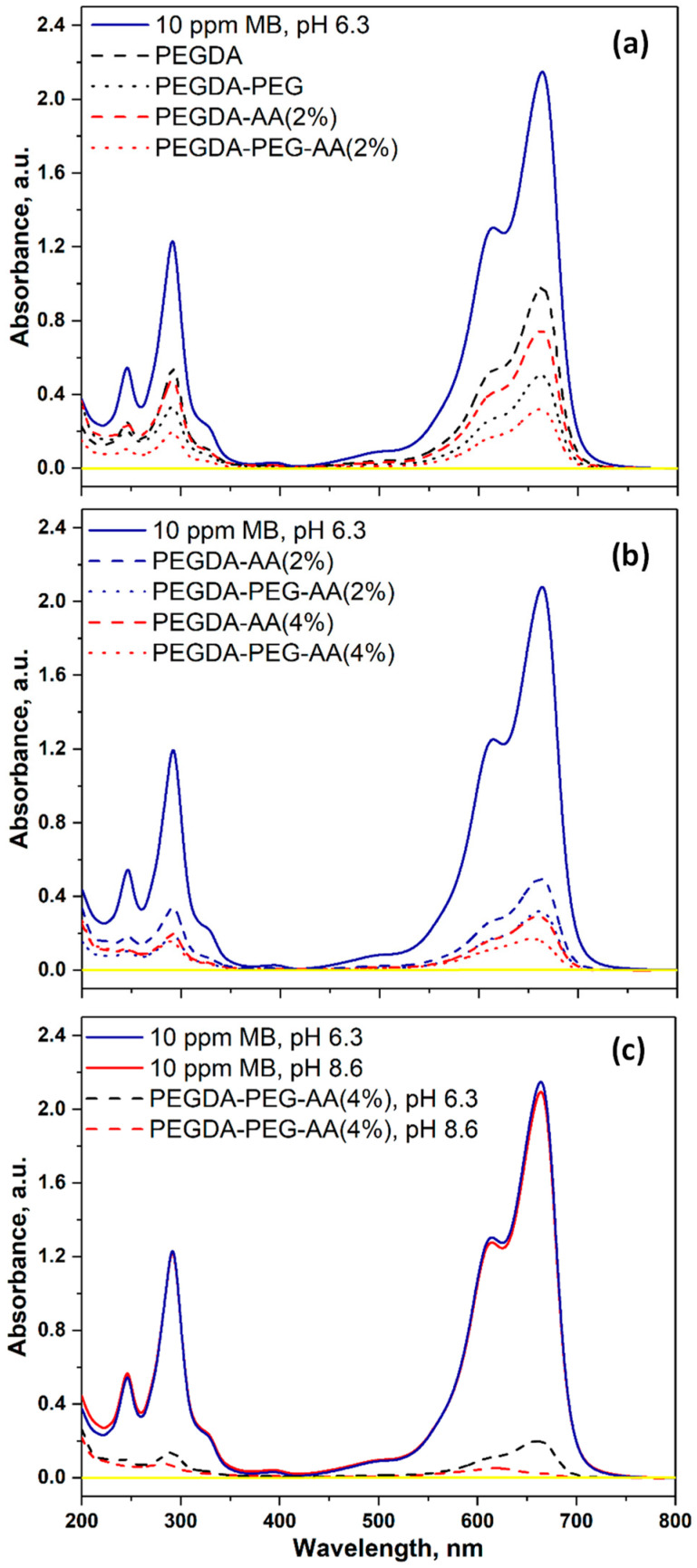
The UV–vis spectra of 10 ppm MB solution before and after equilibrium adsorption onto different microgel samples: (**a**) The comparison between PEGDA and PEGDA/AA microgels; (**b**) The comparison between 2 wt% AA and 4 wt% AA in the dispersed phase; (**c**) The comparison between pH = 6.3 and pH = 8.6. The solution volume was 6 mL and the amount of microgels used was 15.5 ± 1.5 mg for nonporous beads and 12.5 ± 1.5 mg for macroporous beads. The signal for pure water is shown by a yellow line.

Figure 7a shows the UV–vis spectra of 10 ppm MB solution at pH = 6.3 before and after adsorption on various PEGDA and PEGDA/AA microgels. The absorption peak of MB at 664 nm was the highest for the initial 10 ppm MB solution (solid blue line). All microgel samples adsorbed a certain percentage of MB from the solution, leading to a decrease in peak height at equilibrium. The least efficient beads were PEGDA (dashed blue line), since they had a noncharged surface and minimum surface area due to nonporous structure. The most efficient beads were PEGDA–PEG–AA(2%) (dotted red line), due to electrostatic attraction between MB^+^ species and –COO– groups and macroporous structure. The second most efficient beads were PEGDA–AA(2%) (dashed red line), due to their negative surface charge but smaller surface area. 

Figure 7b shows the impact of AA content in the dispersed phase on the UV–vis spectra of the dye solution after adsorption onto PEGDA–AA microgels. At equilibrium, the great majority of MB was removed from the solution and the residual peak for all samples was much smaller than the peak for the initial 10 ppm MB solution. The least efficient sample was PEGDA–AA(2%) (dashed blue line), due to the minimum charge and the minimum surface area. On the contrary, the most efficient sample was PEGDA–PEG–AA(4%) (dotted red line) due to the maximum charge and the maximum surface area. 

Figure 7c shows the impact of pH on the UV–vis spectra of the dye solution after adsorption on PEGDA–AA microgels. The pH in the range of 6.3–8.6 does not have any impact on the UV–vis pattern of the initial MB solution (blue and red solid lines). However, at pH = 8.6, the residual MB peak almost completely disappeared at equilibrium (red dashed line) and was only slightly higher than the background value (yellow line). At pH = 6.3, the MB peak was more prominent (blue dashed line). 

The photographs of the initial MB solution and the MB solution at the adsorption equilibrium with PEGDA–AA microgels at the pH values of 6.3 and 8.6 are shown in Figure S2. 

#### 2.5.2. Comparison of Adsorption Efficiency of Cationic Dye (MB) and Amphoteric Dye (RhB) onto Nonporous PEGDA/AA Microgels

**Figure 8:**
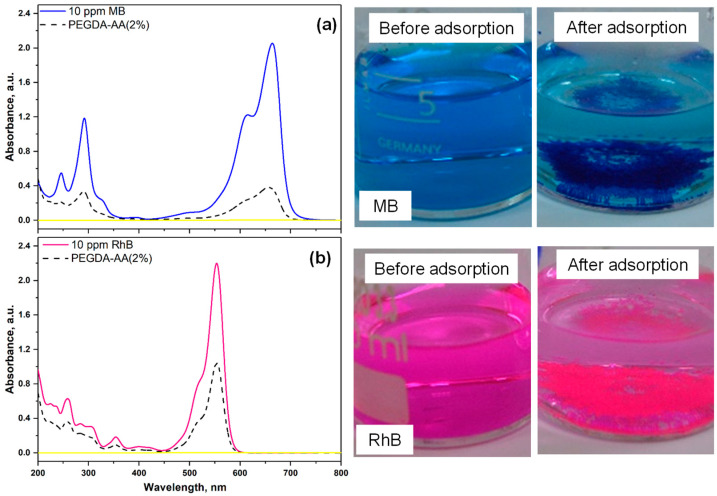
The UV–vis spectra and corresponding photographs of the dye solution before and after 24 h of adsorption onto PEGDA–AA(2%) microgel at pH = 6. The investigated dye was as follows: (**a**) Methylene blue (MB); (**b**) Rhodamine B (RhB). In each case, ~16 mg of the microgel was added into 6 mL of 10 ppm dye solution.

In these experiments, 16 mg of PEGDA–AA(2%) beads were placed into 6 mL of dye solution (10 ppm MB or 10 ppm RhB) at pH = 6 and the suspension was stirred for 24 h. The dye solution, before and after adsorption, was analyzed by UV–vis spectroscopy, and the results for cationic dye (MB) and amphoteric dye (rhodamine B, RhB) were compared in Figure 8. Nonporous PEGDA/AA beads efficiently removed MB from 10 ppm solution due to electrostatic interactions, as can be seen by a significant decrease of the UV–vis absorption peak of MB and decolorization of MB solution after treatment, Figure 8a. In addition, microgel particles that settled at the bottom of the beaker became dark blue. RhB is an amphoteric dye since it contains two cationic amino groups and one anionic carboxylic group. At pH < 5, RhB is present in its cationic form with positively charged amino groups and an uncharged carboxylic group [31]. At pH 5, RhB transforms from a cationic form to a zwitterionic form since amino groups remain positively charged but the carboxylic group becomes negatively charged, so both charges are present in the same molecule. RhB is in its zwitterionic form at pH 6, which is probably the reason for the less efficient adsorption of RhB at pH 6, compared to MB, Figure 8b. RhB binds to PEGDA/AA surface due to electrostatic interactions between negatively charged carboxylic groups of AA residues and positively charged amino groups of RhB. 

The removal efficiencies of both dyes and the corresponding adsorption capacities of the beads were calculated from Equations (2) and (3) and listed in Table 3. The photographs of both particles after dye adsorption are shown in Figure S3a,b. Optical microscopy images of PEGDA–AA(4%) microgel after adsorption of MB from 100 ppm solution at pH 6 are provided in Figure S3c,d, to highlight their strong affinity towards MB. 

As shown in Table 3, under the same experimental conditions, the removal efficiency of MB (cationic dye) was higher than the removal efficiency of RhB (zwitterionic dye). Due to the same reason, the $q_{e}$ value for MB was 3.0 mg/g as compared to 2 mg/g for RhB. 

#### 2.5.3. Adsorption of Anionic Dyes (MO and CR) onto Nonporous PEGDA/AA Microgels

**Figure 9:**
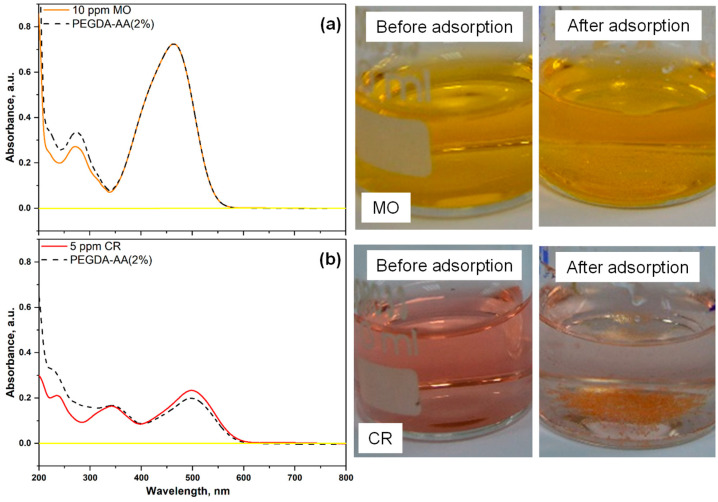
UV–vis spectra and the corresponding solutions of (**a**) methyl orange (MO) and (**b**) congo red (CR), before and after 24 h of adsorption onto PEGDA–AA(2%) microgels. In both cases, ~16 mg of the microgel was added into 6 mL of the dye solution (10 ppm MO or 5 ppm CR at pH = 6). The spectral line for pure water (background signal) is shown by a solid yellow line.

Figure 9 shows the UV–vis spectra of two anionic dyes, methyl orange (MO) and congo red (CR), after their adsorption from 10 ppm MO or 5 ppm CR solutions onto PEGDA–AA(2%) microgel at pH 6. The corresponding E and $q_{e}$ values calculated from Equations (2) and (3) are listed in Table 4. As shown in Figure 9a, the UV–vis absorption spectrum of MO does not show any difference before and after adsorption on PEGDA/AA microgel and therefore, the removal efficiency of MO from its 10 ppm solution was zero, Table 4. In addition, the adsorption capacity was zero, indicating that negatively charged MO molecules are efficiently repulsed by negatively charged bead surfaces. The removal efficiency of CR, another anionic dye, was less than 30% (Table 4), as compared to the removal efficiency of cationic dye MB, which was more than 80% under similar experimental conditions (Table 3). The removal efficiency of CR would be even smaller if the initial concentration of CR in the liquid phase was 10 ppm instead of 5 ppm. 

#### 2.5.4. Adsorption of Copper Cations onto Nonporous and Macroporous PEGDA and PEGDA/AA Microgels

In these experiments, 16 mg of various microgels were placed into a copper nitrate solution (6 mL) containing 50 ppm Cu(II) at a pH of 5.5 or 6.7. The mixture was then stirred over 24 h to achieve an adsorption equilibrium. After that, the equilibrium Cu(II) concentration in the liquid phase was measured, and the corresponding removal efficiency of Cu(II) from the solution and the adsorption capacity of the beads were calculated, Table 5. The maximum pH value that could be tested was 6.7 because copper hydrolysis occurs at pH ≥ 6.8, leading to the precipitation of Cu(OH)_2_. Therefore, it would be impossible to distinguish between the removal of Cu(II) due to adsorption onto microgel particles and due to the precipitation of copper hydroxide. 

As can be seen from Table 5, pure PEGDA beads removed only 6.2% of Cu(II) ions due to the neutral surface and the absence of electrostatic interactions between the microgel surface and Cu(II) ions. PEGDA–AA(2%) beads were better adsorbents for Cu(II) due to electrostatic attraction between Cu^2+^ and negatively charged microgel surface. The removal efficiency of Cu(II) was improved nearly four times when the content of AA in the dispersed phase was increased from 2 wt% to 4 wt%. The maximum adsorption capacity towards Cu(II) was achieved for PEGDA–PEG–AA(4%) beads due to the highest charge and surface area amongst the tested adsorbents. Furthermore, the removal of Cu(II) was much more efficient at pH = 6.7 (E = 98.3%) than at pH = 5.5 (E = 72.9%), due to the higher proportion of charged carboxylic groups at pH 6.7. The results in Table 5 indicate that PEGDA/AA microgels are efficient adsorbents for both organic and inorganic cations.

## 3. Conclusions

Negatively charged, pH-sensitive PEGDA/AA microgels have been synthesized by copolymerizing PEGDA with acrylic acid (AA) within monodispersed emulsion droplets generated in a Lego-inspired glass capillary microfluidic device. AA is a biocompatible, non-toxic monomer that was successfully crosslinked with PEGDA in various ratios to produce microgels with negatively charged surfaces that can interact electrostatically with positively charged molecules at pH > 4. In addition, PEGDA/AA microgels have been found efficient in repelling negatively charged molecules, such as methyl orange and congo red. The surface charge and adsorption properties can be tuned by the AA:PEGDA ratio in the dispersed phase. The higher the content of AA in the monomer mixture, the higher the adsorption capacity towards positively charged species. Furthermore, we have successfully produced macroporous PEGDA/AA microgels with improved adsorption properties by adding non-crosslinkable PEG into the dispersed phase as a porogen and extracting PEG after polymerization with acetone. The resulting macroporous microgels have cylindrical crater-like pores with a size between 1−2 μm and floral topography surrounding the pores due to expulsion of PEGDA through dense polymer skin during leaching. PEGDA/AA microgels showed improved adsorption capacity towards positively charged organic dyes and Cu(II) compared to PEGDA microgels. The ability of charged PEGDA/AA microgels to selectively interact with charged species offers great opportunities to achieve precise control over the loading and delivery of charged bioactive compounds, fouling behavior, and cellular interactions. 

## 4. Materials and Methods

### 4.1. Materials

Poly (ethylene glycol) diacrylate (PEGDA, average MW = 700 g/mol, Sigma Aldrich, St. Louis, MO, USA) and acrylic acid (AA, Thermo Scientific, San Diego, CA, USA) were chosen as a UV-curable macromer and anionic co-monomer. Poly (ethylene glycol) (PEG, average MW = 600 g/mol, Sigma Aldrich) was a porogen, highly compatible with PEGDA but non-crosslinkable, while 2-hydroxy-4′-(2-hydroxyethoxy)-2-methylpropiophenone (Irgacure 2959, Sigma Aldrich) was used as a photo-initiator (PI). XIAMETER^®^ RSN-0749 Resin and XIAMETER^®^ PMX-200 (100 cSt), both supplied by Dow Corning, Midland, MI, USA, were used as a surfactant and oil in the continuous phase. OTMS (octadecyltrimethyoxysilane, Sigma Aldrich) was a hydrophobic coating material for glass capillaries. Acetone (Honeywell) was a solvent for PEG leaching. Cationic dyes, methylene blue (MB, MW = 319.85 g/mol, λ_max_ = 664 nm) and rhodamine B (RhB, MW = 479.02 g/mol, λ_max_ = 554 nm), and anionic dyes, congo red (CR, MW = 696.66 g/mol, λ_max_ = 497 nm) and methyl orange (MO, MW = 327.33 g/mol, λ_max_ = 464 nm), were purchased from Acros Organics (Geel, Belgium). Copper(II) nitrate hemi(pentahydrate) (Cu(NO_3_)_2_·2.5 H_2_O, Sigma Aldrich, USA) was used as a source of Cu(II) ions. All the chemicals were of analytical reagent grade and used without further purification.

Stock dye solutions (1000 ppm) were prepared by dissolving 100 mg of each dye in 100 mL of water, while the stock solution of Cu(II) (1000 ppm) was prepared by dissolving an appropriate amount of Cu(NO_3_)_2_·2.5 H_2_O in water. Working solutions were prepared before each experiment by diluting the stock solutions. The initial pH of the working solutions was adjusted using either 0.1 mol dm^−3^ HNO_3_ or 0.1 mol dm^−3^ NaOH. The pH was measured using Metrohm’s 914 pH/Conductometer (Metrohm, Herisau, Switzerland). All the solutions were prepared using deionized water supplied by a Milli-Q water purification system (Simplicity^®^ Water Purification System, Millipore Corporation, Billerica, MA, USA). 

### 4.2. Glass Capillary Microfluidic Device

Two Lego-inspired blocks (acetal plastic) were designed using SolidWorks software, and were CNC-milled using a HAAS Super Mini mill. The outer capillary (World Precision Instruments, Hitchin, UK, 1.56/2.0 mm inner/outer diameter) of 15 mm length was placed between the two Lego blocks and was immobilized by screws, O-rings, and fasteners. The inner capillary (World Precision Instruments, 0.59/1.0 mm inner/outer diameter) of 152 mm length was pulled with a P-97 Sutter puller and sanded to the orifice size of 350 μm. To prevent wetting by the dispersed phase, the tip was immersed in OTMS and left to dry, providing a hydrophobic surface layer. The inner capillary was inserted into the outer capillary through a tube connector and a hole in one of the Lego blocks and fixed in place with O-rings, screws, and the tube connector. 

### 4.3. Microgel Synthesis

**Figure 10:**
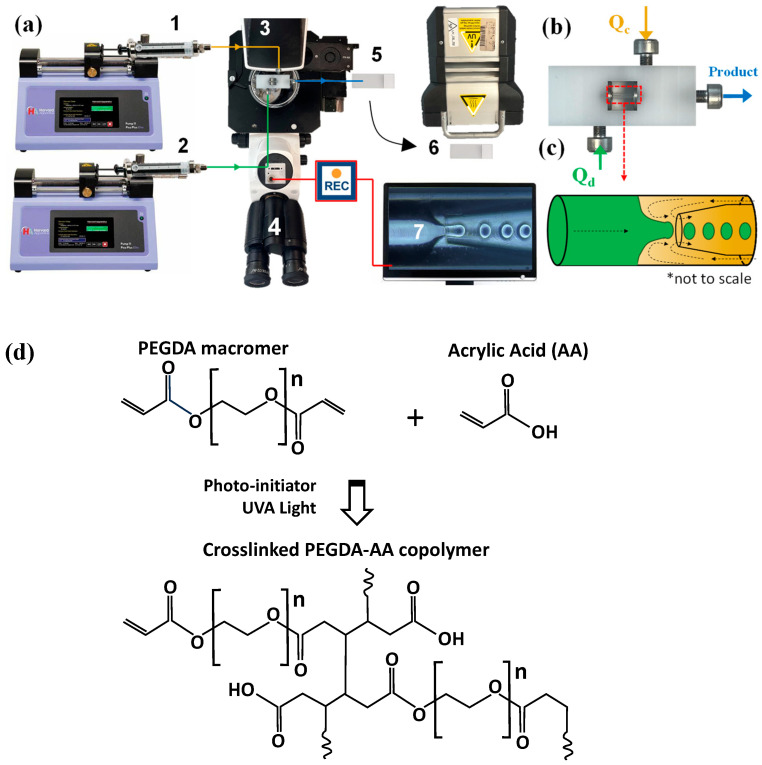
(**a**) Schematic diagram of the microfluidic rig set-up (1: Continuous phase syringe pump, 2: Dispersed phase syringe pump, 3: Lego-inspired microfluidic device, 4: Inverted microscope, 5: Droplet collection, 6: UV light exposure, 7: Image acquisition software; (**b**) Lego-inspired microfluidic device; (**c**) The schematic of droplet formation by 3D counter-current flow focusing; (**d**) The synthesis of PEGDA-AA copolymer network by co-polymerisation of PEGDA and AA.

The dispersed phase solutions used are shown in Table 6. The solutions were mixed with a magnetic stirrer for 1 h to ensure complete dispersion of each component. The dispersed and continuous phases were loaded into SGE 10 mL syringes and supplied to the device with syringe pumps (Harvard Apparatus, 11 Elite) using polyethylene medical tubing (Figure 10a). Any inlet tubes were fixed by stainless-steel tube connectors screwed into the corresponding holes in the Lego device (Figure 10b). The dispersed phase syringe was wrapped in aluminum foil to prevent early polymerization of PEGDA. 

Droplet generation was observed through an inverted microscope using a Basler camera (resolution 728 × 544 pixels, 25 frames per second) and Basler image acquisition software. Droplets were generated within the inner capillary by flow focusing (Figure 10c), collected on a glass slide, and polymerized by placing the slide underneath a UV lamp (UVHAND 250GS, Münster, Germany), Figure 10d. The microgels were washed 3–5 times with acetone and DIW to remove traces of oil. In addition, microgels made with the addition of PEG were suspended in DIW and mixed for one hour. Every 5 min, the solution was replaced with fresh DIW to ensure the full removal of PEG.

### 4.4. Characterisation of Droplets and Particles 

The average droplet/particle diameters were quantified using ImageJ 2.9.0 software. The oval tool was manually placed over 100 contours of the droplets/particles, for which the diameters were measured. 

The surfaces of synthesized particles were characterized using scanning electron microscopy (SEM). Samples were prepared in a Quorum Q150R S, whereby a layer of gold and palladium was deposited on the surface for 60 s. Secondary electrons were measured using a Hitachi TM3030Plus tabletop microscope.

### 4.5. Batch Adsorption Experiments

The experiments were conducted at room temperature by mixing wet microspheres and the dye solution of the specified concentration at pH 6.3. The suspension, in a closed glass vial, was shaken on a laboratory shaker (Promax 2020, Heidolph, Germany) at a constant speed of 150 rpm for 24 h. After that, the liquid phase was separated from the microgels, and the residual concentration of MB in the solution was determined by UV–vis spectrophotometer (UV-2600, Shimadzu, Japan) at the maximum absorbing wavelength of 664 nm. Finally, the microgels were dried in an oven at 60 °C for 24 h and the mass of the dried adsorbent was weighed. 

The effects of different formulations and operating parameters, such as the initial pH (3–8), the acrylic acid (AA) content in the dispersed phase (2–4 wt%), the microgel macroporosity, and the initial MB concentration (1–100 ppm) on the adsorption process were investigated. The dye removal efficiency, E (%), and the equilibrium adsorption capacity, *q_e_* (mg g^−1^) of microgels were calculated using Equations (2) and (3):

(2) $$E=\left(\frac{C_{i}-C_{e}}{C_{i}}\right)100$$

(3) $$q_{e}=\left(\frac{C_{i}-C_{e}}{m}\right)V$$

where $C_{i}$ and $C_{e}$ (mg/L) are the initial and equilibrium concentrations of MB in the solution, respectively, V (L) is the solution volume, and m (g) is the mass of dried microgel.

### 4.6. Equilibrium Analysis

The equilibrium adsorption data obtained by batch experiments were analyzed using the non-linear forms of the Langmuir and Freundlich isotherm models. The Langmuir model assumes that the uptake of dye occurs on a homogeneous surface by monolayer adsorption without any interaction between adsorbed molecules. The maximum adsorption capacity and the Langmuir constant were calculated using the equation:

(4) $$q_{e}=\frac{q_{m}K_{L}C_{e}}{1+K_{L}C_{e}}$$

in which *q_m_* is the maximum adsorption capacity or the amount of dye adsorbed per unit mass of microgel required to cover the microgel surface completely as a monolayer (mg g^−1^) and *K_L_* is the Langmuir constant (L/g), which is related to the adsorption energy. The Freundlich isotherm was developed for multilayer adsorption on a heterogeneous surface, accompanied by interaction between adsorbed molecules, and is given by:

(5) $$q_{e}=K_{F}C_{e}^{1/n}$$

where *K_F_* ((mg/g)/(mg/L)^1/*n*^) and *n* (dimensionless) are the Freundlich constants related to the adsorption capacity and adsorption intensity, respectively [32,33]. The parameters of the Langmuir isotherms (*q_m_* and *K_L_*) and Freundlich isotherms (*n* and *K_F_*) were evaluated by fitting the equilibrium adsorption data to the corresponding isotherm model using the Mathcad software, version 14.0.

## Figures and Tables

**Table 1:** The flow rates of the dispersed phase (DP) and continuous phase (CP) for microgel synthesis, with their respective curing time under UV light (75 mW/cm^2^). Details of dispersed phase compositions are reported in Table 6. The orifice size of the inner capillary was 350 μm.

Sample	DP Flow Rate, Q_d_ (mL/h)	CP Flow Rate, Q_c_ (mL/h)	UV Exposure Time (s)
PEGDA	0.10	0.35	75
PEGDA–PEG	0.13	0.70	300
PEGDA–AA(2%)	0.16	0.45	360
PEGDA–PEG–AA(2%)	0.20	0.55	600
PEGDA–AA(4%)	0.16	0.45	360
PEGDA–PEG–AA(4%)	0.17	0.34	600

**Table 2:** Langmuir and Freundlich parameters for the adsorption of MB dye onto nonporous and macroporous PEGDA and PEGDA/AA microgels. R is the correlation coefficient.

Adsorbent	Langmuir Isotherm			Freundlich Isotherm		
	q_m_, mg/g	K_L_	R	nF	K_F_	R
PEGDA, pH = 6.3	2.9	0.58	0.984	4.6	1.2	0.955
PEGDA–PEG, pH = 6.3	6.4	0.33	0.996	3.5	2.0	0.964
PEGDA–AA(2%), pH = 6.3	8.8	0.19	0.989	2.9	2.1	0.975
PEGDA–PEG–AA(2%), pH = 6.3	15	0.20	0.996	2.8	3.5	0.974
PEGDA–AA(4%), pH = 6.3	15	0.15	0.996	2.6	3.0	0.982
PEGDA–PEG–AA(4%), pH = 6.3	30	0.15	0.993	2.1	5.0	0.967
PEGDA–AA(4%), pH = 8.6	23	0.58	0.991	3.5	8.8	0.991
PEGDA–PEG–AA(4%), pH = 8.6	45	0.21	0.978	3.4	15	0.983

**Table 3:** The removal efficiency (E) and the equilibrium adsorption capacity ($q_{e}$) of cationic (MB) and zwitterionic (RhB) dye from their 10 ppm solutions achieved using PEGDA–AA(2%) beads. The UV–vis spectra and corresponding photographs of the residual solutions are shown in Figure 8.

Dye	E, %	$q_{e}$, mg/g
MB	81.1	3.0
RhB	52.6	2.0

**Table 4:** The removal efficiency (E) and the equilibrium adsorption capacity ($q_{e}$) of anionic dyes (MO and CR) from their respective solutions (10 ppm MO or 5 ppm CR) at pH = 6 using PEGDA–AA (2%) beads. The UV–vis spectra and photographs of the residual dye solutions are shown in Figure 9. The other experimental conditions are shown in the caption of Figure 9.

Dye	E, %	$q_{e}$, mg/g
MO	0	0
CR	29.6	0.7

**Table 5:** The removal efficiencies of Cu(II), E, and the equilibrium Cu(II) adsorption capacities, $q_{e}$, of different microgels at pH = 5.5 and 6.7. The microgel loading in the solution was 2.7 mg/mL.

Adsorbent	pH	E, %	$q_{e}$, mg/g
PEGDA	5.5	6.2	1.1
PEGDA–AA(2%)	5.5	7.0	1.3
PEGDA–AA(4%)	5.5	27.1	3.7
PEGDA–PEG–AA(4%)	5.5	72.2	13.8
PEGDA–AA(4%)	6.7	67.4	11.2
PEGDA–PEG–AA(4%)	6.7	98.3	22.7

**Table 6:** The dispersed phase compositions used to prepare microgel samples (PI = photoinitiator, AA = acrylic acid, PEG = polyethylene glycol, PEGDA = poly (ethylene glycol) diacrylate, DIW = deionized water).

	PI (wt%)	PEGDA (wt%)	AA (wt%)	PEG (wt%)	DIW (wt%)
PEGDA	2	98	-	-	-
PEGDA–PEG	2	78	-	20	-
PEGDA–AA (2%)	2	76	2	-	20
PEGDA–PEG–AA (2%)	2	38	2	20	38
PEGDA–AA (4%)	2	74	4	-	20
PEGDA–PEG–AA (4%)	2	38	4	20	36

## Data Availability

The data presented in this study are available on request from the corresponding author.

## Supplementary Materials

The following supporting information can be downloaded at: https://www.mdpi.com/article/10.3390/gels9110849/s1, Figure S1: PEGDA–PEG–AA (2%) microgel particles suspended in silicon oil observed through an optical microscope after exposure to UV light of 75 mW/m^2^ for (a) 120 s, (b) 240 s, (c) 480 s, and (d) 600 s. Longer UV exposure times resulted in a smoother surface.; Figure S2: Photographs of the initial MB solution (in the middle), and the MB solution after 24 h of adsorption onto PEGDA–PEG–AA(2%) microgel at pH = 6.3 (right) and pH = 8.6 (left); Figure S3: (a,b): Photographs of PEGDA–AA(2%) beads dyed with 10 ppm MB solution (a) and 10 ppm RhB solution (b) at pH 6; (c,d): Optical microscopy images of PEGDA-AA(4%) beads dyed with 100 ppm MB solution at pH 6 taken using 4× objective lens (c) and 2× objective lens (d).
