# Supplementary material for: Selective Adsorption of Ionic Species Using Macroporous Monodispersed Polyethylene Glycol Diacrylate/Acrylic Acid Microgels with Tunable Negative Charge
Source: Gels. 2023 Oct 26;9(11):849. doi: 10.3390/gels9110849 (PMC10670463; doi:10.3390/gels9110849)
Supplement: Supplementary file 1 [file gels-09-00849-s001.zip › gels-2671882-supplementary.pdf]

Supplements

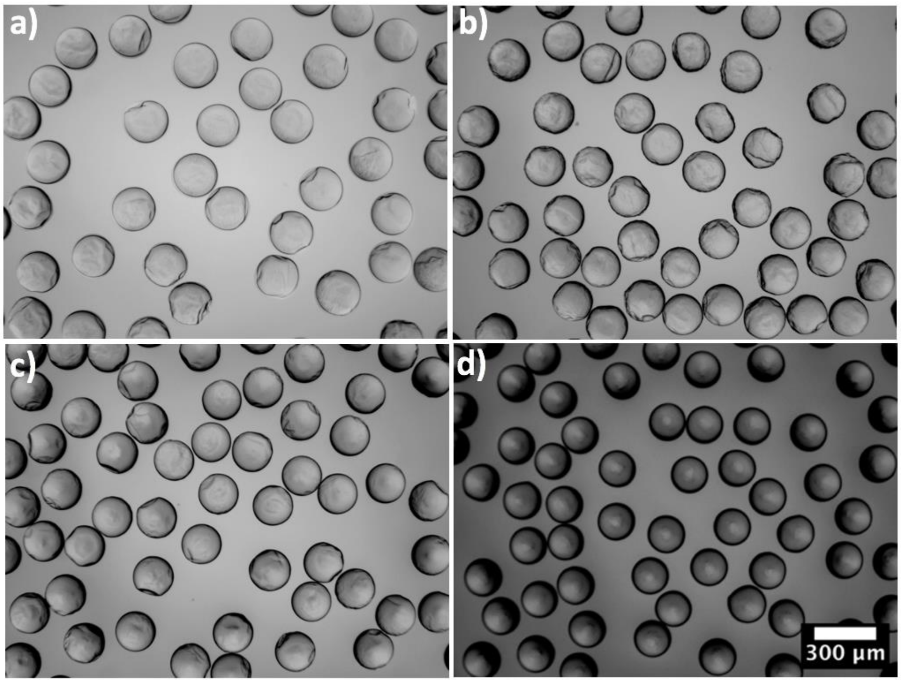

Figure S1. PEGDA–PEG–AA (2%) microgel particles suspended in silicon oil observed through an optical microscope after exposure to UV light of 75 mW/m^2 for (a) 120 s, (b) 240 s, (c) 480 s, and (d) 600 s. Longer UV exposure times resulted in a smoother surface.

Particles gradually shrink during polymerization due to losing internal water and have a wrinkled surface until they become completely polymerized, Figure S1 (a-c). A wrinkled surface occurred due to internal stresses as a result of non-uniform polymerization, with a solid crosslinked core and liquid uncured surface region.

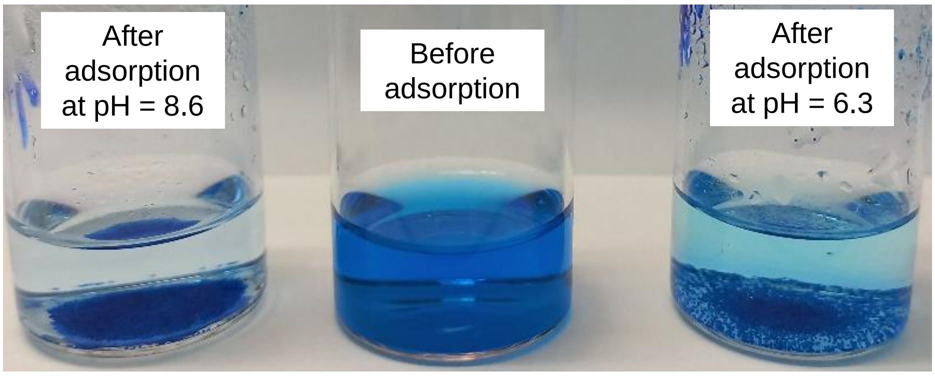

Figure S2. Photographs of the initial MB solution (in the middle), and the MB solution after 24 h of adsorption onto PEGDA–PEG–AA(2%) microgel at pH = 6.3 (right) and pH = 8.6 (left).

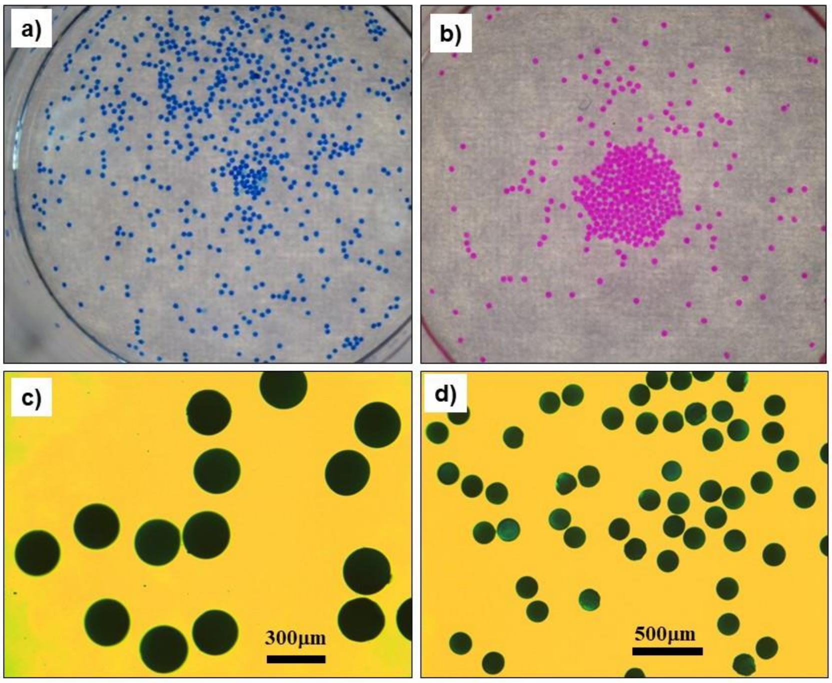

Figure S3. **(a)-(b)**: Photographs of PEGDA–AA(2%) beads dyed with 10 ppm MB solution **(a)** and 10 ppm RhB solution **(b)** at pH 6; (c)-(d): Optical microscopy images of PEGDA–AA(4%) beads dyed with 100 ppm MB solution at pH 6 taken using 4× objective lens **(c)** and 2× objective lens **(d)**.

PEGDA–AA(4%) beads dyed with 100 ppm MB are much darker than PEGDA–AA(2%) beads dyed with 10 ppm MB due to the higher charged particles and much higher concentration of the initial MB solution.
